# A comparison between IBEX bone health applied to digital radiographs and dual-energy X-ray absorptiometry at the distal-third and ultra-distal regions of the radius

**DOI:** 10.1186/s12891-024-07670-0

**Published:** 2024-07-24

**Authors:** Ben Lopez, Robert Meertens, Mike Gundry, Paul Scott, Mr Ben Crone, Richard McWilliam

**Affiliations:** 1 Research and Development, Ibex Innovations Ltd, Explorer 2, Netpark, Sedgefield, County Durham TS21 3FF UK; 2 St Luke’s Campus, University of Exeter, Exeter, EX1 2LU UK

**Keywords:** Opportunistic screening, DXA, aBMD, T-score, Forearm, Wrist, Osteoporosis

## Abstract

**Background:**

In an ageing population, low impact fragility fractures are becoming increasingly common. However, fracture risk can be reduced where low bone density can be identified at an early stage. In this study we aim to demonstrate that IBEX Bone Health (IBEX BH) can provide a clinically useful prediction from wrist radiographs of aBMD and T-score at the ultra-distal (UD) and distal-third (DT) regions of the radius.

**Methods:**

A 261-participant single-centre, non-randomised, prospective, study was carried out to compare a) IBEX BH, a quantitative digital radiography software device, to b) Dual-energy X-ray Absorptiometry (DXA). A total of 257 participants with wrist digital radiograph (DR), forearm DXA pairs were included in the analysis after exclusions.

**Results:**

The adjusted R^2^ value for IBEX BH outputs to the radial areal bone mineral density (aBMD) produced by a GE Lunar DXA system for the UD region is 0.87 (99% Confidence Interval (CI) [0.84, 0.89]). The adjusted R^2^ value for IBEX BH outputs to aBMD for the DT region is 0.88 (99% CI [0.85, 0.90]). The Area Under the Receiver Operating Characteristic curve (AUC) for the forearm T-score ≤  − 2.5 risk prediction model at the UD region is 0.95 (99% CI [0.93, 0.98]). The AUC for the forearm T-score ≤  − 2.5 risk prediction model at the DT region is 0.98 (99% CI [0.97, 0.99]).

**Conclusion:**

From a DR of the wrist, IBEX BH provides a clinically useful i) estimate of aBMD at the two regions of interest on the radius and ii) risk prediction model of forearm T-score ≤  − 2.5 at the UD and DT regions.

## Background

The global ageing population and associated increase in the risk of osteoporotic fractures pose significant socioeconomic challenges for healthcare systems and the wider economy [1]. 1 in 3 women over the age of 50 years and 1 in 5 men are predicted to experience osteoporotic fractures within their lifetime [2]. Along with a high prevalence, there exists significant number of individuals who would benefit from treatment for osteoporosis but do not receive it. For example, a 66% treatment gap was reported in 2019 for women in the United Kingdom (UK) [3]. Therefore an opportunity exists to narrow the treatment gap resulting in fewer osteoporotic fractures in the population, improved patient outcomes and lower healthcare costs.

In the UK, the National Osteoporosis Guideline Group (NOGG) recommends [4] use of the validated Fracture Risk Assessment Tool (FRAX) [5]. A FRAX assessment can be made based on clinical risk factors alone, as the typical first step to determine whether the patient should be put directly onto treatment or whether referral for further investigations is warranted. This would typically include a Dual-energy X-ray Absorptiometry (DXA) scan to measure areal Bone Mineral Density (aBMD).

The NOGG guidance indicates that the assessment should be conducted for patients with a clinical risk factor for fragility fracture [4], for example, post-menopausal over 50 s. Since a fragility fracture is reported to increase the risk of a subsequent fracture by 1.86 (95% confidence interval (CI) [1.75, 1.98]) [6], Fracture Liaison Services (FLSs) have been tasked with reducing secondary fractures. This has resulted in the majority of fracture risk assessments being triggered by a fragility fracture. FLSs have proven effective as a cost effective means of reducing fractures [7]. However, key performance indicators are not related to primary prevention [8] and hence there is less incentive to identify and treat patients for osteoporosis that have not experienced a fracture. This situation is symptomatic of the predominately reactionary approach to osteoporosis care in the UK, with 98% of the total economic burden being allocated to fracture care and the remaining 2% to identification and treatment of osteoporosis [3].

Increasing primary prevention would be beneficial—not least to the patient whose fracture risk can be substantially mitigated following diagnosis [9]—but also to the healthcare provider through the reduced cost of treatment prior to fracture [10, 11]. One method to increase primary prevention is the IBEX Bone Health (IBEX BH) software, which provides a measure of forearm aBMD and forearm T-score at the ultra-distal (UD) and distal-third (DT) regions of the radius [12, 13] from standard digital radiographs (DRs) of the wrist. The software provides an incidental finding alongside the diagnostic image with no additional dose to the patient and no impediment to radiographers.

Although Neck Of Femur (NOF) and Lumbar Spine (LS) DXA are more often used for clinical decision making, distal radius aBMD is predictive of fracture risk. The risk ratio (RR) of fracture increases by 1.4 (95% CI [1.3, 1.6]) for every one standard deviation (SD) decrease in aBMD at the distal radius [14]. The risk ratio of 1.4 implies a threshold can be set for aBMD at the distal radius such that the cohort of patients with aBMD below this threshold will have fracture risk as high as those that have already had a fracture. Therefore, measuring aBMD at the forearm would enable a group of patients to be identified whose risk level is comparable to those targeted by FLS, but who are currently not picked up by the health service. IBEX BH measured at the forearm therefore opens the possibility for a wider screening provision via fast and readily available DR systems as a mass screening tool, or an opportunistic screening approach for patients already presenting to radiology for a forearm X-ray.

This paper reports the results of a study comparing IBEX BH to the clinical standard DXA as a tool to measure forearm aBMD at the UD and DT regions.

## Materials and methods

### Device description and technical background

The IBEX BH software extracts additional information from a standard DR (X-ray image) using a fully automated data pipeline. A physics model of the X-ray system is built and used to efficiently simulate the X-ray image output for a given body part ([13] Chapter 4). This physics model is calibrated to an X-ray system at a particular kV ([13] Chapter 6). Inputs to the simulator that describe the body part comprise two maps: one map describes the Posteroanterior (AP) total thickness of the arm and the other describes the proportion of hard and soft tissues (referred to here as alloy). A mapping of bone thickness is produced by multiplication of the thickness map by the alloy map such that, when multiplied by the assumed density of bone, becomes aBMD. The simulation is then compared to the real X-ray image and the error between simulated and real images are used to refine the model by making morphological changes to the two maps. In this inverse problem solving approach, the comparison is repeated many times for varying total thickness and alloy, until the simulated X-ray image and the real X-ray image agree within the underlying statistical errors of the system ([13] Chapter 5). At this point, the algorithm is deemed to have derived an acceptable match and the resulting model of the body part provides an accurate representation of ground truth morphology. This model can then be interrogated to infer aBMD, which can finally be transformed into a T-score using population data.

Derivation of bone density via standard single energy X-ray exposure has, until now, not been possible owing to degeneracy occurring between the measured X-ray intensities and the object’s true density and thickness; it is possible for two objects of different densities and different thicknesses to exhibit the same level of X-ray absorption when imaged at a single beam energy [15]. DXA resolves this degeneracy by acquiring data at multiple energies while IBEX BH takes the alternative approach of exploiting the morphological model of the object and its composition-dependent X-ray scattering properties. The foundational assumption being made is that: in the presence of scatter, all anatomically plausible solutions for soft and hard tissue distribution— for which agreement can be found between the physics model, morphological model and the ground truth—exhibit similar aBMD properties to the ground truth of the actual forearm being imaged ([13] Chapter 7).

When used as an opportunistic screening tool the software must run without impeding clinical workflow and hence human intervention must be minimised. This is achieved by automating identification of the region of interest (ROI) to be measured, a process that typically requires manual intervention by the user interpreting a DXA scan. Automated ROI selection involves a process of tissue-bone segmentation and key point analysis. The tissue-bone segmentation algorithm uses a proprietary convolutional neural network (CNN) trained with 1158 hand labelled X-ray images of multiple body parts. The ROI detection algorithm uses a region-based CNN trained on 1622 hand-labelled wrist radiographs to segment the radius and the ulna. Image processing techniques are then used to identify the styloid process of the radius which is used as a key point to define the UD and TD ROIs. No data from participants enrolled in this study were used in the training of these algorithms.

**Fig. 1:**
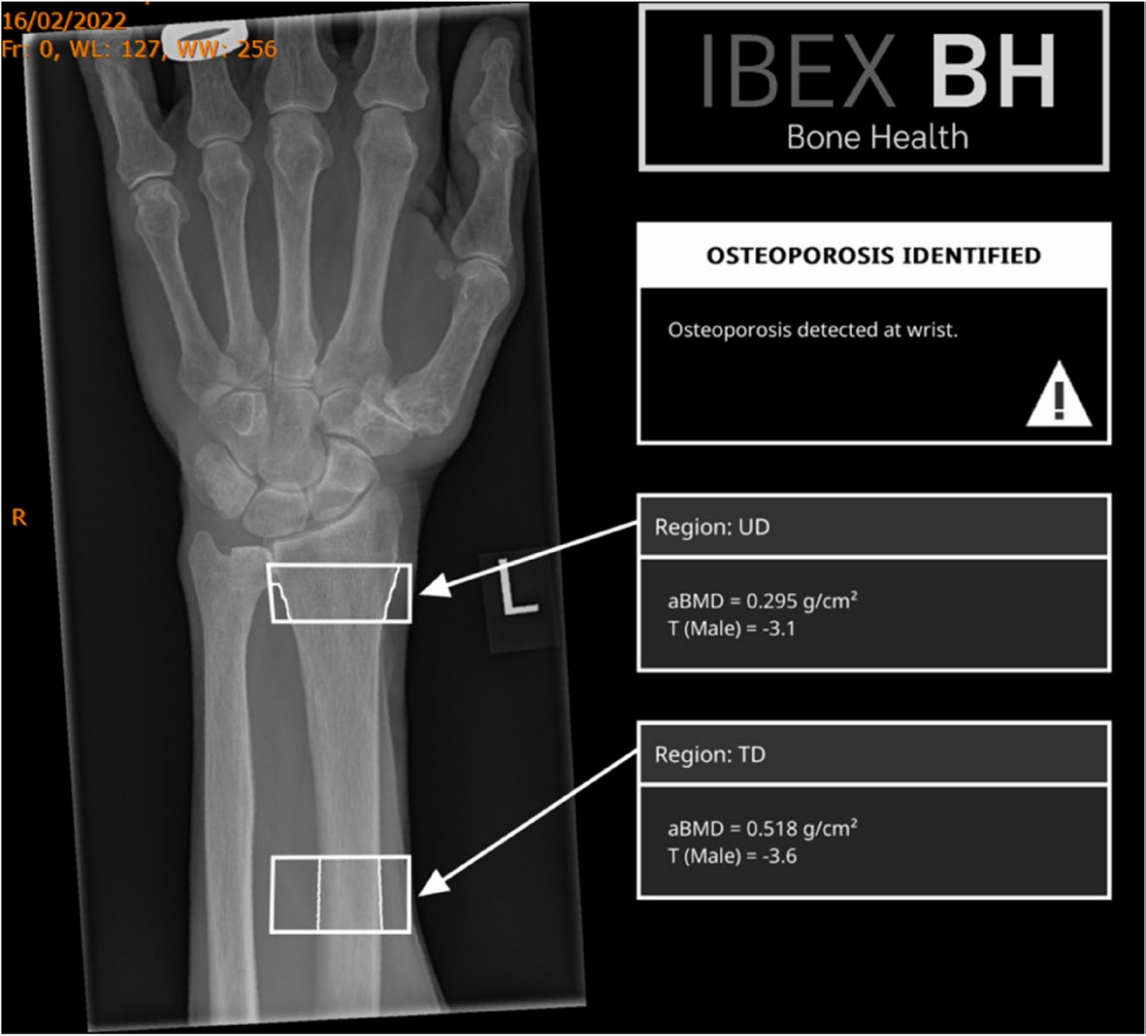
IBEX Bone Health DICOM report displaying the ultra-distal (UD) region and distal-third regions of interest with their associated forearm aBMD and T-score estimates

Figure 1 shows an output DICOM from the IBEX BH software showing the post processed image, both UD and DT ROI and the associated aBMD and T-scores.

### Enrolment

A 261-participant single-centre, non-randomised, prospective, study was carried out in order to compare IBEX BH to DXA, where DXA is considered a reference standard as the most common clinical method of assessing osteoporosis [16]. Recruitment was performed with the target population between February and October 2022. Participants meeting the following criteria were considered eligible for the study:

1. Male or female, 50 years of age or over.
2. Either:
  a member of the group of 10,000 individuals pre-consented by Exeter University to for participation in clinical research,
  attending radiology department for plain radiograph of PA forearm or wrist showing UD radius,
  outpatients with a scheduled DXA assessment,
  other members of the public who wished to participate.

3. Able to comprehend and sign the Informed Consent prior to enrolment in the study.

Participants meeting the following criteria were not eligible for the study:

1. women who are pregnant or are breastfeeding,
2. participants who have previously sustained a fracture that affects the UD radius on both arms,
3. participants who have sustained fractures in both hips,
4. have implants or other radio-opaque objects in the region of study,
5. unwilling or unable to provide informed consent.

### Data collection

All participants underwent a DXA scan to determine aBMD at both forearms (UD and TD radius). All aBMD measurements were performed on the same DXA scanner throughout (GE Lunar Prodigy Advanced). Five qualified radiographers of varying experience carried out the DXA measurements. Precision and accuracy were monitored daily using a manufacturer-supplied phantom. All quality assurance checks were within the manufacturer’s tolerances throughout the study. All DXA images were reported on by a qualified DXA reporting radiographer; an Associate Professor in MSK Imaging. Where appropriate, ROIs were manually repositioned to correct for errors in DXA’s automated ROI selection algorithm. Outputs were reported in two forms: i) assuming that the patient’s arm length (measured as the distance from the tip of the ulnar styloid to the olecranon) had been measured and ii) using the DXA default of 29 cm as the patient’s arm length. This approach accounted for the two major reporting styles for DXA forearms in clinical practice.

Participants also received two PA wrist radiographs (both left and right wrists) using an AGFA DR100s mobile DR system. IBEX BH software was calibrated to the DR system at 60kVp at the beginning of the study using a set of phantoms. Additional weekly calibration data sets were collected for potential re-calibration of the software, but this was not required since system drift was observed to be within acceptable limits. The following exposure parameters were fixed for all DR acquisitions: field of view = 24 cm × 12 cm, source voltage = 60kVp and current = 2mAs. Four independent qualified radiographers of varying experience carried out DR measurements. All DR and DXA images were reported on by a reporting radiographer and sent to the patient’s GP but their reports are not used in this study.

Participants completed a questionnaire to record aspects of their medical history. The patient’s age, sex, height, weight and clinical fracture risk factors were recorded. The clinical risk factors were whether: i) the patient had a previous fracture, ii) their parent had a fractured hip, iii) they are a current smoker, iv) they are taking glucocorticoids, v) they have rheumatoid arthritis, they have secondary osteoporosis and vii) they drink 3 or more units of alcohol per day.

Two types of derived ROI data were input to the IBEX BH software. The first used manually segmented images and ROIs manually matched to those of DXA. This assesses the current upper bound to performance, given user intervention. The second approach utilised full automation available within the software wherein no user intervention is required. Here both segmentation and ROIs are derived automatically. This case assesses the more likely clinical use scenario for which full automation is required. The analysis was repeated for both DXA report types (default 29 cm and the participant’s arm length) to enable detailed comparison with the two most prevalent DXA reporting protocols.

### Statistical analysis

Continuous patient demographic factors are reported as the sample mean and sample SD of i) the entire cohort, ii) the cohort defined by DXA forearm T-score ≤  − 2.5 at the UD or DT region using the reports with the participants arm length (forearm osteoporosis) and iii) the cohort defined by DXA forearm T-score >  − 2.5 at a UD and a DT region (forearm non-osteoporosis). To assess differences between the forearm osteoporosis and the forearm non-osteoporosis cohorts for these factors, test statistics for a t-test and a Wilcoxon signed rank test [17] are reported. Test statistics are reported in place of *p*-values since the majority of *p*-values are < 0.001. The Area Under the Receiver Operating Characteristic curve (AUC) [18] for classifying the forearm osteoporosis cohort for each variable is also reported. A value of 0.5 indicates no discrimination and a value of 1 indicates perfect discrimination of the DXA result. DXA and IBEX BH outputs are reported in the same way as continuous patient demographic factors except that the analysis was done per forearm rather than per patient.

Categorical patient demographic factors are reported as the prevalence in the i) entire cohort, ii) forearm osteoporosis cohort and iii) forearm non-osteoporosis cohort. A *p*-value under the null hypothesis that the prevalence is the same for the forearm osteoporosis and the forearm non-osteoporosis cohort is also reported.

To assess the performance of IBEX BH as a predictor of forearm aBMD, a multi-variate linear regression analysis was performed, in which all outputs from the IBEX BH software—total thickness (cm), alloy and bone thickness (cm)—were analysed together to predict forearm aBMD. All three Trueview outputs were added to model selection as there was a statistically significant difference between the forearm osteoporosis and forearm non-osteoporosis cohort. A forward–backward stepwise model selection with the Akaike information criterion was used to select the models presented [19]. The base model had linear and quadratic terms for every variable included. We report the adjusted *R*^2^ value of the resultant models with a 99% CI estimated using bootstrapping with 5000 samples [20]. The analysis is done independently for each ROI (UD and DT), for each DXA reporting protocols (patient’s arm length and 29 cm arm length) and each IBEX BH version (manual or automatic ROIs).

To assess the performance of IBEX BH as a risk predictor of forearm osteoporosis at the UD and DT regions, logistic regression risk prediction analysis was performed on forearm T-scores derived from the predicted forearm aBMD. Predicted forearm aBMD is transformed to a forearm T-score by the following equation.

1 $$\mathrm T\;\mathrm{Score}\;=\frac{\mathrm{aBMD}\;-\;\upmu}{\mathrm\sigma}$$

where µ is the sex-specific mean of a healthy population and σ is the sex-specific standard deviation of a healthy population. To assess the quality of the risk prediction model, ROC analysis was performed. The ROC curve and the AUC with associated 99% CI by De long’s method [18] is reported.

To assess fracture risk identification, sensitivity, and specificity of both DXA forearm T-score ≤ -2.5 at either the UD or DT regions and IBEX BH forearm T-score ≤ -2.5 at either the UD or DT regions for two cohorts with and without historical fractures is calculated. Sensitivity and specificity are reported with 99% CIs.

No missing data imputation was required in this analysis. The statistical analysis was performed with the statistical software package, R [21].

## Results

Results from the 261 eligible participants enrolled in the study are reported here. 3 participants were excluded owing to their forearm DXA scans being rejected by the reporting radiographer since they were not commensurate with the trial protocol. 1 participant’s DR header files were corrupted so the trial protocol could not be confirmed and therefore excluded. The number of participants after exclusions reported in our analysis is therefore 257. 11 participants had DXA only on the left arm and 11 had DXA only on the right arm. 1 further participant had DR only on the left arm and 1 further had DR only on the right arm. DXA and DR scans were not performed on 1 arm due to: jewellery that could not be removed; a previous injury with a dressing still present and a previous fracture at the UD region. The total number of forearms with DXA scans reported in our study is 492. The number of wrists with DR scans included in the analysis is 490. Overall, 1.53% of participants and 6.13% of wrists were excluded from the analysis.

Table 1 reports the continuous demographic risk factor summary statistics. Table 2 reports the categorical demographic and risk factors summaries. Table 3 summarises the DXA and IBEX BH outputs. Table 4 reports the linear regression models mapping IBEX BH outputs to DXA forearm aBMD adjusted R^2^ Values.

**Table 1:** Table of continuous variables split by i) all and ii) forearm osteoporosis (O) defined as forearm T-score ≤  − 2.5 at either left or right ultra distal region, as measured by DXA with participant’s arm length. The total number of participants reported in this table is *n* = 257 (80 in the forearm Osteoporosis Cohort). The mean of the cohort and the standard deviation (SD) of the cohort are reported in the first three columns. The test statistics of a t-test and a Wilcoxon signed rank test for the difference between forearm osteoporosis and forearm non-osteoporotic cohorts are reported in the fourth and fifth columns. All T-test Statistics and Wilcoxon test statistics were statistically significant with a *p*-value < 0.001. The sixth column reports the area under the receiver operating characteristic curve (AUC) for classifying the forearm osteoporosis cohort

	Mean all (SD)	Mean O (SD)	Mean non-O (SD)	T	W	AUC
Age (years)	70.92 (9.03)	73.88 (8.27)	69.75 (9.12)	3.52	8007.5	0.62
Height (cm)	167.93 (9.74)	162.16 (8.41)	170.55 (9.17)	-7.20	3509.50	0.75
Weight (kg)	72.35 (14.34)	62.57 (11.67)	76.78 (13.21)	-8.68	2713	0.81

**Table 2:** Demographic factors split by i) all and ii) forearm osteoporosis (O) defined as forearm T-score  ≤−2.5 at either left or right ultra distal region, as measured by DXA with participant’s arm length. The total number of participants reported in this table is *n* = 257 (80 in the forearm Osteoporosis Cohort). Column one reports the number of participants without that factor. Column two reports the percentage of the entire cohort without that factor. Column three reports the number with that factor in the forearm osteoporotic cohort. Column four is the percentage with the factor in the forearm osteoporotic cohort. Column five reports the number with that factor in the forearm non-osteoporotic cohort. Column six is the percentage with the factor in the forearm non-osteoporotic cohort. Column seven is the *P*-value testing whether the percentages are different

	Total	%	n O	% O	n Non-O	% Non-O	*P*-value
Male (No)	144	56.03	70	87.50	74	41.81	< 0.001
Previous Fracture (No)	237	92.22	68	85	169	95.48	0.01
Parental hip fracture (No)	220	85.60	69	86.25	151	85.31	0.99
Smoker (No)	254	98.83	79	98.75	175	98.87	1
Gluccocorticoids (No)	237	92.22	71	88.75	166	93.78	0.25
Rhuematoid arthritis (No)	246	95.72	76	95	170	96.05	0.96
Secondary osteoporosis (No)	215	83.66	61	76.25	154	87.01	0.05
High alcohol use (No)	227	88.33	76	95	151	85.31	0.04

**Table 3:** Table of continuous DXA and IBEX BH variables split by i) all and ii) forearm osteoporosis (O) defined as forearm T-score ≤  − 2.5 at ultra distal or distal-third region, as measured by DXA with participants arm length. The total number of participants reported in this table is n = 257. 492 DXA forearms are reported and 490 (134 in forearm osteoporosis cohort) IBEX BH wrists are reported. IBH auto is the IBEX BH using the automated ROI selection, IBH actual uses the matched ROIs to DXA with participants arm length and IBH actual matched ROIs to DXA with default 29 cm arm length. The mean of the group and the standard deviation (SD) of the group are reported in columns one to three. The test statistics of a t-test and a Wilcoxon signed rank test for the difference between forearm osteoporosis and forearm non-osteoporotic groups are reported in the fourth and fifth columns. All t-tests and Wilcoxon tests were statistically significant with *p* < 0.001. The sixth column reports the area under the receiver operating characteristic curve (AUC) for classifying the forearm osteoporosis group

	Mean all (SD)	Mean O (SD)	Mean non-O (SD)		T	W	AUC
DXA aBMD DT	0.66 (0.12)	0.51 (0.07)	0.720	(0.09)	-27.48	1292	0.97
DXA aBMD UD	0.35 (0.09)	0.25 (0.04)	0.389	(0.06)	-28.67	846	0.98
DXA aBMD DT 29 cm	0.67 (0.12)	0.52 (0.07)	0.724	(0.08)	-26.57	1441	0.97
DXA aBMD UD 29 cm	0.35 (0.09)	0.25 (0.04)	0.391	(0.06)	-29.00	796	0.98
IBH Auto DT Bone Thickness	1.54 (0.24)	1.27 (0.14)	1.640	(0.18)	-23.56	2614	0.95
IBH Auto UD Bone Thickness	1.03 (0.17)	0.85 (0.09)	1.096	(0.14)	-23.02	3078	0.94
IBH Auto DT Thickness	6.34 (0.61)	5.87 (0.45)	6.584	(0.54)	-14.80	7360	0.85
IBH Auto UD Thickness	4.59 (0.50)	4.10 (0.32)	4.776	(0.43)	-18.90	4828	0.90
IBH Auto DT Alloy	0.76 (0.03)	0.78 (0.03)	0.749	(0.03)	11.48	37,868	0.79
IBH Auto UD Alloy	0.78 (0.03)	0.79 (0.02)	0.771	(0.02)	10.28	36,329	0.76
IBH Actual DT Bone Thickness	1.56 (0.23)	1.29 (0.14)	1.66	(0.18)	-24.09	2494	0.95
IBH Actual UD Bone Thickness	1.01 (0.17)	0.83 (0.09)	1.08	(0.14)	-22.59	3300	0.93
IBH Actual DT Thickness	6.16 (0.68)	5.55 (0.50)	6.39	(0.59)	-15.80	6564	0.86
IBH Actual UD Thickness	4.58 (0.51)	4.09 (0.32)	4.77	(0.44)	-18.92	4878	0.90
IBH Actual DT Alloy	0.75 (0.03)	0.77 (0.03)	0.74	(0.03)	9.42	35,846	0.75
IBH Actual UD Alloy	0.78 (0.02)	0.80 (0.03)	0.77	(0.02)	10.56	36,540	0.77
IBH 29 DT Bone Thickness	1.54 (0.24)	1.27 (0.15)	1.64	(0.18)	-23.28	2690	0.94
IBH 29 UD Bone Thickness	1.01 (0.17)	0.83 (0.09)	1.08	(0.15)	-22.63	3376	0.93
IBH 29 DT Thickness	6.37 (0.60)	5.86 (0.45)	6.57	(0.54)	-14.63	7567	0.84
IBH 29 UD Thickness	4.58 (0.51)	4.09 (0.32)	4.77	(0.44)	-18.93	4892	0.90
IBH 29 DT Alloy	0.78 (0.03)	0.78 (0.03)	0.75	(0.03)	11.44	37,750	0.79
IBH 29 UD Alloy	0.78 (0.02)	0.80 (0.02)	0.77	(0.02)	10.48	36,402	0.76

**Table 4:** Table reporting the adjusted R^2^ values for linear regression models mapping IBEX BH to DXA forearm aBMD with 99% confidence intervals. IBEX BH Manual uses ROIs manually matched to DXA while IBEX BH auto uses the automated ROI selection. The first column reports the values matching to DXA reports that used the patient’s arm length. The second column reports the values matching to DXA reports with 29 cm arm length

	DXA Actual R^2^ [LCI, UCI]	DXA 29 cm R^2^ [LCI, UCI]
IBEX BH Manual UD	0.87 [0.83,0.89]	0.86 [0.83,0.89]
IBEX BH Manual DT	0.88 [0.85,0.90]	0.88 [0.86,0.91]
IBEX BH Auto UD	0.87 [0.84,0.89]	0.87 [0.83,0.90]
IBEX BH Auto DT	0.87 [0.85,0.90]	0.89 [0.86,0.91]

**Fig. 2:**
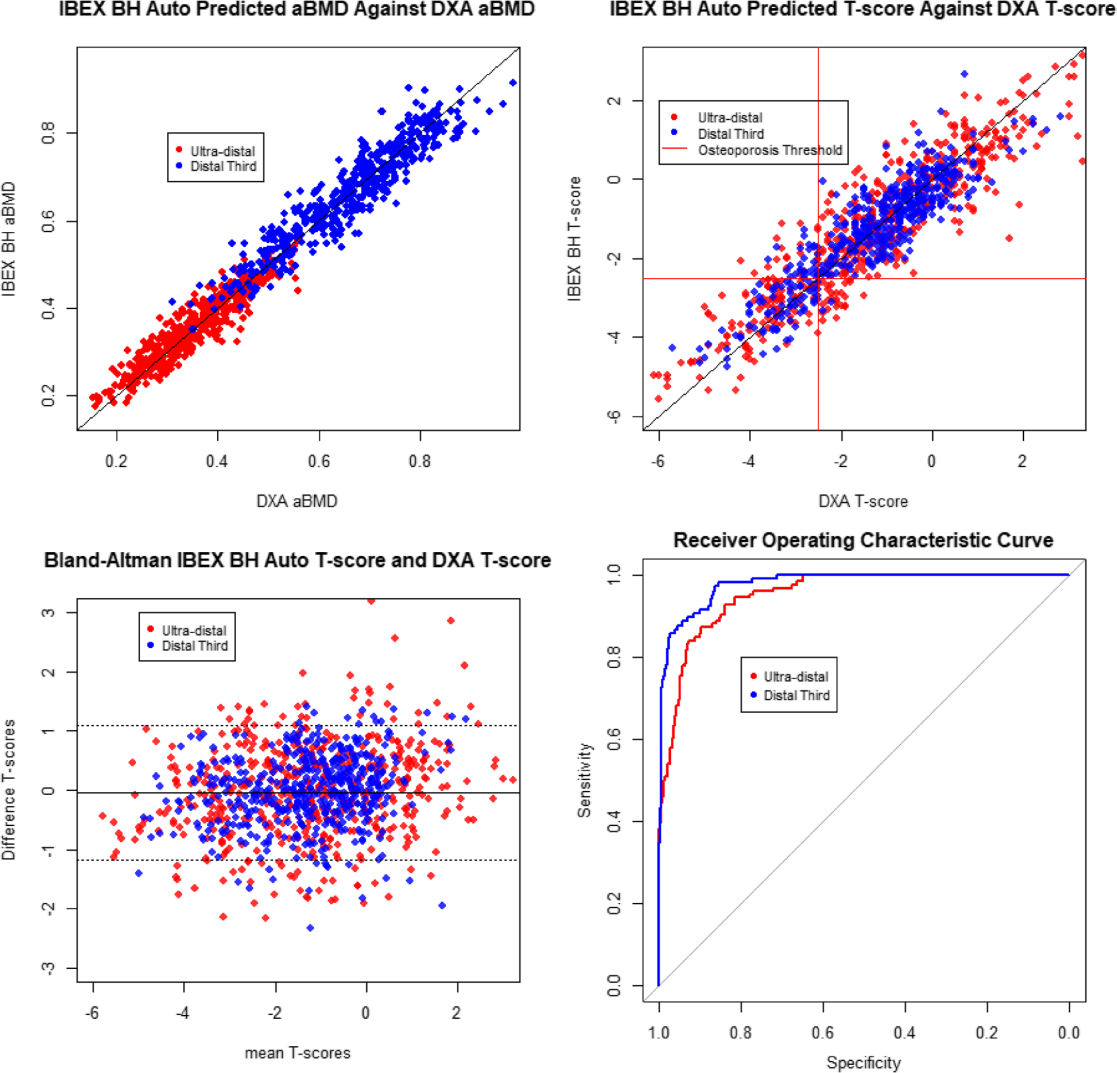
Top left: IBEX Bone Health predicted aBMD using the automated software against DXA forearm aBMD reported using the participant’s arm length at the ultra-distal and distal-third regions. Top right: IBEX Bone Health predicted forearm T-score using the automated software against DXA forearm T-score reported using the participant’s arm length at the ultra-distal and distal-third regions. The manufacturer’s reference ranges were used to calculate the forearm T-score. Bottom left: Bland–Altman plot depicting the difference between DXA forearm T-scores using a participant’s arm length and IBEX BH forearm T-scores using the automated software at the ultra-distal and distal-third regions. Bottom right: Receiver Operating Characteristic curve of the output of the logistic regression model prediction DXA (reported with the patient’s arm length) forearm T-score ≤  − 2.5 using the automated IBEX BH outputs

Figure 2 top left shows the predicted forearm aBMD plotted against DXA forearm aBMD for the automated IBEX BH approach and DXA reported with the patient’s actual arm length. The standard deviation of the residuals is 0.042. Figure 2 top right shows the predicted forearm T-score plotted against DXA forearm T-score for the automated IBEX BH approach and DXA reported with the patient’s actual arm length. Figure 2 bottom right shows a Bland-Altmann plot comparing DXA forearm T-score and the automated IBEX BH approach.

The AUC for the forearm osteoporosis risk prediction model at the UD region is 0.95 (99% CI [0.93, 0.98]). The AUC for the forearm osteoporosis risk prediction model at the DT region is 0.98 (99% CI [0.97, 0.99]). The ROC curves are shown in Fig. 2 bottom right. There is no statistically significant difference between the AUC for the UD and DT regions at the 99% confidence level.

The sensitivity and specificity of DXA forearm T-score ≤ -2.5 to historical fracture is 0.60 (99% CI [0.30, 0.85]) and 0.71 (99% CI [0.63, 0.79]) respectively. The sensitivity and specificity of IBEX BH forearm T-score ≤ -2.5 to historical fracture is 0.49 (99% CI [0.37, 0.62]) and 0.73 (99% CI [0.66, 0.80]) respectively.

## Discussion

Table 4 indicates a strong relationship between IBEX BH and DXA, with all adjusted R^2^ values between 0.86 and 0.89. The correlation between IBEX BH and the GE Lunar DXA system is 0.932 which is also within the bounds of the reported correlations between different DXA manufacturers [0.78, 0.95] [22]. These results indicate that the software performs at a similar level to commercially available DXA systems and should therefore be above the performance level required for a clinically useful screening device. This is further supported by the AUCs (0.95 for the UD radius and 0.98 for the DT radius) for discriminating whether the T-score ≤  − 2.5 at that ROI. Using this classifier, patients at high risk of osteoporosis at the wrist could be identified with clinically useful sensitivity and specificity.

Table 4 also shows no statistically significant difference between the adjusted R^2^ values reported for manual and automated use of IBEX BH, indicating that the automation features within the software are non-inferior to manual user intervention. This offers the possibility of avoiding human placement error associated with differences in ROI placement, the need for user training and enables the software to be integrated with minimal impediment to radiology workflow. These results indicate that the software could be used as an opportunistic screening tool with standard DR procedures.

The least significant change of IBEX BH could not be measured as the study design did not allow for multiple images to be taken of the same patient. The residual standard deviation of 0.042 provides an analogous measure to the least significant change reported on the forearm DXA reports 0.016. This implies the difference between IBEX BH and DXA is above intra-machine variability but within inter-machine variability.

Table 1 displays results for the continuous demographic factors. As expected, the older the participant the lower their forearm aBMD and the heavier a participant the higher their forearm aBMD. Weight is most predictive of a forearm T-score ≤  − 2.5 at the UD or DT regions with an AUC of 0.81 compared to age which is the least predictive with an AUC of 0.62. Table 2 displays results for the categorical demographic factors. Sex is the strongest predictor of forearm T-score ≤  − 2.5 with females more likely to have a forearm T-score ≤  − 2.5.

Table 3 displays results for the continuous DXA and IBEX BH outputs. DXA is most predictive of forearm T-score ≤  − 2.5 with a minimum AUC of 0.97 compared to a maximum AUC of 0.95 for IBEX BH outputs. This is expected as DXA was used to define the forearm osteoporosis and forearm non-osteoporosis cohorts so it should have the highest AUC. The AUC is not 1 for DXA since sex was not included and forearm osteoporosis at the forearm was defined using T-scores. IBEX BH bone thickness is the most predictive of forearm T-score ≤  − 2.5, followed by total thickness and finally alloy. This is expected since, if the same bone material assumptions were used and there was no error in IBEX BH or DXA, bone thickness and DXA aBMD would theoretically have correlation equal to 1.

Comparing Tables 1 and 3, all DXA outputs, IBEX BH bone thickness and IBEX BH total thickness are more predictive than the continuous demographic factors. IBEX BH alloy is more predictive than age and height but not weight. The total thickness is the next best predictor as there is a high correlation between the total AP thickness and the thickness of the bone and it is a better predictor at the UD region as there is less variability in the tissue surrounding the UD region than there is in the DT region. The conclusions from this table are that all IBEX BH outputs appear to be predictive of DXA and therefore are justified for inclusion in the subsequent model selection. The differences in predictive ability make intuitive sense given the meaning of IBEX BH outputs and forearm morphology.

An alternative to IBEX BH as an opportunistic screening tool in DR is radiogrammetry, which looks at geometric features of the bone like cortical thickness to infer forearm aBMD. Whilst cortical thickness is linked to bone strength, there is evidence to suggest that cortical changes can occur as a result of ageing independently of aBMD [23]. The correlation to a number of DXA devices was reported between 0.72 and 0.83 [22] which is lower than the lowest correlation 0.93 achieved here. It is hypothesised that this is because IBEX BH uses a physics-based inverse problem solving approach that solves the same fundamental problem as DXA: that a dense bone and a porous bone can exhibit equivalent intensity values in a radiograph depending on the surrounding tissue. Radiogrammetry measures a distinct quantity and relies on its correlation to DXA forearm aBMD. These results indicate that IBEX BH outperforms radiogrammetry in comparison to the reference standard DXA.

Another alternative imaging modality is based on quantitative computed tomography (QCT) scans [24]. At the forearm QCT had a correlation to forearm DXA.

comparable to the results reported here, between 0.82 and 0.93. It can also measure aBMD at the more commonly measured central sites, spine and NoF. However, in the UK the number of DRs examinations is larger than CT (21.4 million compared to 6.6 million [25]), and hence a larger fraction of the target population is accessible via the DR imaging modality. Therefore, IBEX BH has the potential to have wider impact than QCT.

IBEX BH also exceeds the performance reported for a commercially available quantitative ultrasound (QUS) elective screening device which demonstrated an inferior correlation to DXA distal radius and distal tibia aBMD of between 0.61 and 0.71 [26]. Radiofrequency Echographic Multi Spectrometry is another elective screening device that reports a correlation to DXA forearm aBMD of 0.93 [27] which is not significantly different to the results presented here although is at the more clinically relevant site. Whilst it is not possible to make a direct comparison with other devices from this study design, IBEX BH performance is not worse than these elective screening devices in clinical use.

Where a fracture or previous fracture prevents the use of the UD region, this study has shown that the DT provides an effective alternative, (*R*^2^ = 0.88). The DT region is the forearm site most commonly reported by DXA (possibly due to lower fracture incidence) [28]. Initial testing also indicates similar performance may be possible on the metacarpals which have been evidenced by other methods such as Digital X-ray Radiogrammetery to provide clinically useful indications of bone health [23]. Therefore, if a fracture is suspected, a small change to the field of view (increase to 24 cm × 12 cm for example) would enable the software to still assess bone health in the presence of a fracture. Furthermore, further development is likely to also include the metacarpals in which case no adjustment to the field of view would be required.

There is no evidence of a statistically significant difference between ability to identify historical fracture between DXA and IBEX BH. There is some evidence for both IBEX BH and DXA that the specificity is greater than 0.5. There is no evidence for both IBEX BH and DXA that the sensitivity is greater or less than 0.5. Furthermore, due to the low prevalence of historical facture in the study (8%), the tests have low power. Therefore, the study has not shown direct statistical evidence of the ability of IBEX BH to identify individuals at risk of fragility fracture. The intent of his study was to show similarity to a reference standard DXA, which has been shown to help identify fragility fractures before the occurrence of a fracture [14]. This implies that IBEX BH could also be informative of fragility fracture risk.

This study was performed on a single system, at a single kV, at a single mAs, at a single FOV and a single SID. This was because it was intended as an initial demonstration of performance at ideal conditions. As long as the system is linear in mAs, as was the case for tested system, mAs variability is normalised by dividing through by mAs. This is performed automatically in the software to match the calibration mAs and test mAs. Theoretically, differences in kV and systems are taken up by changes to physics model parameters (for example, pixel pitch, kV, source filtration) and the calibration procedure by which the physics model is matched to the system [13]. SID can be corrected mathematically if known or using the part of the image not containing the wrist. FOV theoretically should not impact performance provided the UD and DT regions are present and are not adjacent to the collimator. While these features of clinical variation have been considered by the underlying methodology encoded within the software, further clinical testing is required to prove their effectiveness.

IBEX BH has also been applied to the pelvis in a previous study [12], which is a standard site for diagnosing osteoporosis [29]. A further study is planned (IRAS study reference 326,406) which will assess performance at a clinically relevant patient dose (the previous study having been conducted at one fifth of standard dose). This, combined with improvements to the underlying algorithm is expected to result in improved AUC for osteoporosis diagnosis using IBEX BH.

In conclusion, IBEX BH matches the reference standard DXA at the UD and DT ROIs to a level that suggests that the software would serve as a clinically useful automatic opportunistic screening tool.

### Limitations

The study was carried out at a single centre by a small research team. A single imaging system was used with a fixed protocol so the variation in forearm positioning and acquisition parameters is likely to have been significantly smaller than clinical practice. Further multi-centre clinical studies are needed to evidence that these results can be achieved in clinical practice. A single DXA system was used for the reference standard measurements and as there are differences between manufacturers’ T-scores, for example due to a difference in reference ranges, these results may not transfer directly to other DXA manufacturers.

The study did not directly assess risk of fragility fracture for the IBEX BH outputs, neither retrospective nor prospective. Due to the selection criteria, there were not enough historical fractures for a statistically meaningful analysis. Furthermore, it is not clear whether historical fractures were in fact fragility fractures. A further study comparing historical facture and no fracture cohorts for both IBEX BH and DXA would further increase evidence for IBEX BH’s clinical utility. A prospective study comparing IBEX BH and DXA’s ability to predict fracture would yield the highest level of evidence.

As reported in Table 2, the sample population varies from the target population (over 50 s) with a bias towards i) females, ii) over 70 s and iii) low body mass indexes. Furthermore, the use of a volunteer population means that it is likely fewer participants exhibited co-morbidities relative to clinical practice. Most pertinently, there were no fractures present in any of the forearms analysed. Further clinically-based studies are needed to evidence that these results can be achieved on the target population.

### Further research

1. The data in this study could be used to extend the number of ROIs that could be used. The metacarpals are used in DXR to measure bone health and so may well extend the number of images that IBEX BH can be used on. Significant labelling time is required to train the automated ROI detection for the metacarpals.
2. A follow on study is being undertaken to extend IBEX BH to other body parts for opportunistic screening. These are: ankle, knee and pelvis. Wider compatibility will enable a larger fraction of the target population to be assessed.
3. Further studies are needed to evidence i) the repeatability of the software over time, ii) performance across different system manufacturers, kVs, mAs, FOVs and source imaging distances, and iii) the performance on a wider patient demographic including ethnic minorities.
4. Clinical trials investigating IBEX BH in clinical practice are needed that measure not only its performance against DXA but also its impact on patient outcomes and healthcare costs. This would involve testing a new care pathway wherein high risk participants are referred by IBEX BH for follow up investigation. Ideally, participants would be followed up on for 5–10 years after measurement to compare IBEX BH and DXA’s ability to predict fracture.
5. A retrospective study comparing historical facture and no fracture cohorts for both IBEX BH and DXA.
6. Finally, studies are being considered to extend IBEX BH to mammography systems. In this case an additional scan would be taken when a patient receives their routine breast cancer screening scan. Dose and cost effectiveness become a more complex proposition for elective rather than opportunistic scans. However, the patient demographic is ideally suited to benefit from such provision.

## Data Availability

The data that support the findings of this study are available from the corresponding author, Dr Robert Meertens, upon reasonable request.

## Abbreviations

AUC: Area Under the Receiver Operating Characteristic curve
aBMD: Areal Bone Mineral Density
CI: Confidence Interval
CNN: Convolutional neural network
DR: Digital Radiograph
DT: Distal-Third
DXA: Dual-energy X-ray Absorptiometry
FLS: Fracture Liaison Services
FRAX: Fracture Risk Assessment Tool
IBEX BH: IBEX Bone Health
LS: Lumbar Spine
NOF: Neck of Femur
PA: Posteroanterior
QCT: Quantitative Computed Tomography
ROI: Region Of Interest
NOGG: The National Osteoporosis Guideline Group
UD: Ultra-Distal
UK: United Kingdom
